# A Review of Commercial *Metarhizium*- and *Beauveria*-Based Biopesticides for the Biological Control of Ticks in the USA

**DOI:** 10.3390/insects13030260

**Published:** 2022-03-05

**Authors:** Cheryl Frank Sullivan, Bruce L. Parker, Margaret Skinner

**Affiliations:** Entomology Research Laboratory, University of Vermont, Burlington, VT 05405, USA; bparker@uvm.edu (B.L.P.); mskinner@uvm.edu (M.S.)

**Keywords:** entomopathogenic fungi, microbial biopesticide, biological control, ticks, *Beauveria*, *Metarhizium*, pesticides

## Abstract

**Simple Summary:**

Microbial biopesticides containing entomopathogenic fungi have potential in tick management. In this review, we compiled a comprehensive list of the use of commercialized *Metarhizium* and *Beauveria*-based biopesticides in the USA that have been tested against ixodid ticks under laboratory and field conditions and when used as a part of integrated tick management. Despite considerable progress in the development of fungal biopesticides over the past 20 years, the establishment of commercial products available for use against ticks continues to be slow. There is a need for the development of sustainable, nonchemical tick management strategies. Until efficacious fungus-based products become more available, tick management will rely primarily on synthetic chemical acaricides, with natural-product acaricides as the alternative.

**Abstract:**

There is a need for the development of sustainable, nonchemical tick management strategies. Mycoacaricide and mycoinsecticide product development worldwide has focused primarily on fungi in the genera *Beauveria* (Hypocreales: Cordycipitaceae) and *Metarhizium* (Hypocreales: Clavicipitaceae). Microbial biopesticides containing entomopathogenic fungi have potential in tick management. However, despite considerable progress in the development of fungal biopesticides over the past 20 years, the establishment of commercial products available for use against ticks continues to be slow. We reviewed published scientific literature and compiled a comprehensive list of reports of the effectiveness of commercial biopesticides based on the fungal genera *Metarhizium* and *Beauveria* and registered for use in the USA against ixodid ticks under laboratory and field conditions. We also report on results when these biopesticides were used as a part of integrated tick management. Until efficacious fungus-based products become more available, tick management will rely primarily on synthetic chemical acaricides, with natural-product acaricides as the alternative.

## 1. Introduction

Ticks transmit the greatest diversity of pathogenic organisms of medical and veterinary importance among arthropods affecting the health of humans and domestic and wild animals [1]. Worldwide, they cause billions of dollars in annual losses to the livestock industries and in costs for the diagnosis and treatment of tick-borne illnesses [2,3]. Broadcast applications of acaricides (synthetic, natural-product, or biologically based) is the primary strategy used to reduce the abundance of host-seeking ticks [4]. Synthetic chemical acaricides are the most commonly used, and will continue to be until alternatives with comparable efficacy are found that combat acaricide resistance, particularly in *Rhipicephalus* spp., satisfy consumer interest in ecologically based products, and reduce nontarget effects on other organisms like pollinators [5,6,7,8,9,10].

Entomopathogenic fungi have greater potential to reduce tick densities compared with other biological control agents like parasitic wasps, pathogenic nematodes, and generalist predators such as birds and beetles [2,10,11,12,13]. Mycoinsecticides and mycoacaricides containing entomopathogenic fungi as the active ingredient can be used to suppress arthropods of economic, medical, and veterinary importance when used as an augmentative biocontrol strategy within an integrated pest (tick) management (IPM/ITM) approach [11,14,15,16,17]. Their development and use has been widely studied, with management success against numerous invasive and native arthropod pests and vectors of disease agents in forest, agricultural, and residential settings since the first documented field attempt using mass-produced fungi in 1888 [18,19,20,21,22]. Globally, and in the USA specifically, commercialization has focused primarily on fungi in the genera *Beauveria* (Hypocreales: Cordycipitaceae) and *Metarhizium* (Hypocreales: Clavicipitaceae) [14,16,23].

Mycoacaricide use against ticks is a fairly recent technology in the timeline of biopesticide use, with only two dozen reports worldwide prior to the turn of the 21st century [24]. In 2006, only a few field studies had been carried out in the USA [10,12,13], both targeting the blacklegged tick, *Ixodes scapularis* (Say) [25,26]. A wide breadth of management techniques has focused on this species with the goal of protecting human health from diseases associated with exposure to pathogens the ticks may harbor, particularly *Borrelia burgdorferi* sensu stricto, the causal agent of Lyme disease [7,14,27,28]. The most recently compiled worldwide list of mycoinsecticides and mycoacaricides identified three fungus-based products labeled for ticks [14]. Two products were registered in the USA. At the time of writing, both are unavailable on the market. Despite product availability limitations, several tick species of concern have been targeted with commercial formulations in laboratory trials, field settings, and within an ITM approach. Herein, we review published scientific literature and present a comprehensive list of reports of the effectiveness of commercial fungal biopesticides based on the genera *Metarhizium* and *Beauveria*, registered for use in the USA, and tested against ixodid ticks. The results that are presented are from reports of product applications or applications of fungal strains that were isolated directly from the products.

## 2. *Metarhizium*-Based Formulations

Two isolates of *Metarhizium brunneum* (Petch), formerly *M. anisopliae* (Metschnikoff) Sorokin, have been used as the active ingredient in products used against ixodid ticks: strains F52 and ESC1, hereafter referred to as *Mb*-F52 and *Mb*-ESC1, respectively [7,14,23]. The most recently available strain, *Mb*-F52, was first isolated from codling moth (*Cydia pomonella*) in Austria. The strain has several names globally, including ATCC90448, ARSEF1095, ARSEF7711, Ma43, BIPESCO5, and BIO1020 [29]. It has a wide host range and is commonly used to target thrips (Thysanoptera), vine weevils (Coleoptera), and mites (Acari) in field and greenhouse crop production and ticks in turf/residential lawns, and has few negative impacts on nontarget arthropods when used within label guidelines [29,30,31,32]. From 2008 to 2020, two products were available in the USA for use against ticks under the tradename Met52^®^, formerly Tick-Ex^®^, (Novozymes Biological, Franklinton, NC, USA). The formulations consisted of rice-based granules (Met52^®^G) for broadcast application to turf or incorporation into soil, and an oil-based, emulsifiable concentrate (Met52^®^EC) for foliar spray or soil drench applications. Over the years, market availability has been inconsistent due to reformulation, rebranding, and market consolidations [14,23,33].

Since 2002, most studies using *Mb*-F52 and *Mb*-ESC1 have targeted *I. scapularis*. (Table 1, Table 2 and Table 3). Formulations can cause mortality in all mobile life stages under laboratory, residential yard, and woodland conditions at a variety of application rates. In the laboratory, application rates ranging from 10^6^–10^9^ conidia/unit volume or area generally cause 70–100% adult and nymph mortality within three to four weeks at 23–25 °C and ~90%RH (Table 1). The fungus has also been tested in combination with the synthetic pesticide permethrin, which did not hinder the ability of the fungus to cause mortality [34]. Product tests in the laboratory on other tick species—*Dermacentor albipictus* (Packard), *Dermacentor reticulatus* (Fabricius), *Ixodes ricinus* (L.), *Rhipicephalus sanguineus* sensu lato, and *Rhipicephalus* (*Boophilus*) *microplus* (Canestrini)—also demonstrated high mortality under high temperature and humidity conditions (>80%RH) (Table 4). *Dermacentor albipictus* larvae, a one-host tick that parasitizes ungulates like cattle, horses, and moose [35,36,37] has received the most attention. Although 100% mortality is achievable in vitro after three weeks when immersed or sprayed, broadcast applications of a granular formulation achieved a reduced level of mortality (89%). Under seminatural conditions in the laboratory (containers with sand and a nylon rod for ticks to quest on), mortality for this species was influenced by whether larvae were aggregated in a quiescent state or actively questing, and by the fungal formulation with which they were challenged during their off-host period [38].

With the exception of *Amblyomma americanum* (L.) [42], field applications of *Mb*-F52 products focused on suppressing *I. scapularis* nymphs during their questing period (primarily April–May through August) in the northeastern USA (Table 2 and Table 3). When *M. brunneum* products were applied in the field alone, mixed results were observed (Table 2). A 10 and 41% knockdown control was observed when using *Mb*-F52 in a woodland setting [41], and up to 36% control with *Mb*-ESC1 [34]. One study examined the use of nesting material treated with *Mb*-ESC1 to target larvae that were infesting mice to reduce nymph abundance on the landscape the following year [39]. Although tick mortality of 75% was reported in treated nests versus 35% in control nests in the laboratory, treatments did not have an impact on nymphal densities or the percentage of nymphs infected with *B. burgdorferi*; however, when data were standardized with the long-term averages, nymphal densities were significantly lower in localized areas surrounding the treated nest boxes. In contrast, one study reported that spray applications of *Mb*-F52 resulted in 56 and 85% fewer ticks on lawn and woodland plots, respectively, within one season, whereas in the two previous years, the synthetic chemical pesticide bifenthrin, a pyrethroid, provided 86 and 87% control. The *Mb*-F52 sprays resulted in an 8.6% risk of an infected tick bite compared to 30% in the controls [44]. Repeat applications of fungal products are needed for sustained tick suppression. One report showed only short-term suppression of *I. scapularis* nymphs below a suppression threshold set at 90% [42]. Reapplications of *Mb*-F52 were made every two to three weeks to provide suppression, whereas one early-season application of the chemical pesticide bifenthrin provided 100% suppression. Low residual effectiveness using one application of *Mb*-F52 (<2 weeks) was also observed in a different study [41].

Integrated tick management uses a combination of strategies such as host reduction/treatments, habitat manipulation, and least-toxic pesticides (i.e., fungal biopesticides) targeting different tick life stages as well as their hosts [17,55,56]. Single interventions to manage ticks are often limited in their time to effectiveness, duration, and efficacy [47]. Because of the complex ecology of ticks and tick-borne diseases, single interventions are likely not enough to provide sustained tick suppression that will reduce the risks of pathogen exposure, while ITM has greater potential to overcome these issues [17]. Fungal biopesticides are commonly used as part of the IPM of agricultural and forest pests [19,22] and there is interest in their inclusion in ITM for residential landscapes. When *Mb*-F52 was used in combination with other interventions for the management of *I. scapularis*, reductions in host-seeking ticks generally ranged from 50 to 95% (Table 3). Questing nymphs were reduced by 78–95% at a woodland–lawn edge when *Mb*-F52 was used along with deer reduction and fipronil-based rodent bait boxes [46]. It was also determined that larvae infesting the white-footed mouse, *Peromyscus leucopus*, a primary host, were reduced by as much as 94% in residential neighborhoods that received the treatments [47]. A reduction of up to 53% in the possibility of encountering a questing nymph infected with a pathogen was observed when *Mb*-F52 and fipronil bait boxes were used in combination [45]. These studies demonstrate the effectiveness of an integrated approach to managing *I. scapularis* using *Mb*-F52 as a component to help reduce the risk of encountering pathogen-infected nymphs.

Several reports of *Mb*-F52 use under both laboratory and field conditions have focused on spray applications of oil-based formulations. Spray applications of oil-based formulations generally have superior efficacy against ticks while protecting conidia from adverse environmental effects such as ultraviolet radiation exposure and desiccation, maintaining viability [2,57]. Spray applications targeting foliage/turf where ticks quest have been extensively studied because of the increased potential for tick contact with infective conidia, a critical factor for successful fungal infection [11]. Granule/pellet formulations applied in their solid form also show effectiveness against ticks and there has been recent interest in the development of *Metarhizium*-based types, particularly for one-host species like *D. albipictus* and *R. microplus*, which infest large ungulates [58,59,60,61]. Granules are formulated as an aerial conidia-based material grown on a nutritive substance (i.e., grains) or from microsclerotia (hyphal aggregates) or blastospores produced in liquid culture [59,61,62]. Because granules target the soil/duff layer, the fungi’s natural environment, and have persistence potential [63], they can provide sustained suppression over time, reducing the need for repeat applications. Examinations of the use of *Mb*-F52 granules for ticks are limited to laboratory trials. Two studies examined the use of conidia-based commercial products [38,40]. One of the studies exposed *I. scapularis* to granular *Mb*-F52 and mortality among nymphs ranged from 30.2 to 81.5% after four weeks in the laboratory [40]. Mortality was variable likely due to the nonuniform distribution of the coarse-textured rice, where ticks either contacted a large dose of conidia or missed contact. The other study observed similar results against *D. albipictus* larvae, with mortality ranging from 79 to 82% after three weeks [38]. In a follow-up study, a *Mb*-F52 granular material was formulated using millet, a finer material, and mortality of ~80% was observed with no difference in mortality among application rates [61]. To overcome environmental effects and prolong longevity, prototype granular microsclerotia formulations using *Mb*-F52 have also been tested, with results providing up to 56 and 74% mortality for unfed and fed *I. scapularis* nymphs, respectively, within seven weeks [64]. The ticks were treated with granules that provided 6.3–4.3 × 10^8^ conidia/cm^2^ which persisted over time and remained viable for eight weeks. These are concentrations that would presumably cause mortality to ticks that come into contact with infective conidia. Field efficacy studies using prototype formulations are lacking and should be encouraged.

## 3. *Beauveria*-Based Formulations

The use of commercial strains of *Beauveria bassiana* (Balsamo-Crivelli) Vuillemin against ticks has been investigated to a lesser extent than the use of *Metarhizium*. Reports of the use of these strains are increasing, presumably due to their wide market availability [16]. There are currently five strains formulated as active ingredients (GHA, ATCC 74040, ANT-03, HF-23, and PPRI 5539). They fall under several product lines and are formulated mostly as concentrated suspensions, wettable powders, emulsifiable concentrates, and oil dispersions, primarily with conidia as the infective propagule [16,23]. Evaluations against *I. scapularis* have focused on strain GHA (ARSEF 6444, ATCC74250), referred to as *Bb*-GHA hereafter, the active ingredient in the BotaniGard^®^ and Mycotrol^®^ (LAM International, USA) product lines (Table 3). Strain GHA has also been tested against *A. americanum, D. albipictus, D. reticulatus, I. ricinus, R. sanguineus*, and *H.* *lusitanicum*, primarily under laboratory conditions (Table 5). The strain was first isolated from a chrysomelid beetle in the USA. It is a popular biopesticide that is used in a variety of agricultural, landscape, and turf settings to manage a diversity of pests, with few published nontarget effects [16,65]. Currently, no *B. bassiana* products specifically labeled for ticks are available in the USA.

Like *M. brunneum* products, the efficacy of products using *Bb*-GHA has been variable under both laboratory and field conditions. In the laboratory, *A. americanum* adults treated with *Bb*-GHA survived 7.2 days compared to 17.9 days in the control and the strain caused noticeable desiccation, an effect of fungal infection [66]. When immersed in up to 10^8^ conidia/mL, 100% mortality occurred after three weeks in the European species *I. ricinus* and *D. reticulatus* [53]. Over 90% mortality within four weeks has been observed in aggregated *R. sanguineus* sensu lato [54]. This species is commonly found indoors in dog kennels and homes and is known to be permethrin resistant and tolerant to fipronil, which are commonly used for its control [69]. In contrast, only 30–41% mortality after three weeks occurred in *D. albipictus* larvae that were sprayed with 1 × 10^6^–10^8^ conidia/mL, respectively [52].

*Beauveria bassiana* strain GHA has been shown to cause significant mortality and reduce tick abundance under arena and field conditions. In an arena experiment with cages placed within a forested area in which *Bb*-GHA was tested against *A. americanum,* there was significantly greater mortality among adult ticks recovered from treated arenas compared to untreated control arenas [67]. In Spain, when rabbit (*Oryctolagus cuniculus*) burrows were sprayed with *Bb*-GHA to manage *Hyalomma lusitanicum* Koch and other associated ticks, on-host tick numbers were reduced in spring by 79% and 63% by day 30 and 60, respectively, and in summer by 36% on day 30 [68]. Applications of *Bb*-GHA and *Bb*-ATCC (Naturalis^™^) to lawn–woodland perimeters and residential woodlands for *I. scapularis* nymphs in combination with a wood chip ground barrier was shown to reduce tick abundance on the landscape [44]. In the first year, a reduction of nymph populations was 89% when used with the barrier and 74% without. In the second year, nymph reductions were 55% for both treatments. The probability of encountering a *B.-burgdorferi*-infected tick was 28.5 and 25.3%, which was lower or comparable to sites treated with the synthetic chemical pesticide bifenthrin (37.7 and 24.3%) for each of the two years. Further research using *B. bassiana* products against ticks is warranted given their potential to cause mortality. Given the accessibility of *B. bassiana* products on the market, reports of efficacy testing against ticks will presumably increase in the future until a *Metarhizium*-based product becomes consistently available.

## 4. Factors to Consider

Fungal biopesticides reduce tick abundance via direct mortality or through sublethal effects that reduce fecundity [12,17,70]. Ticks vary greatly in susceptibility to fungal infection based on several factors including their species, life stage, engorgement status, and the fungal species, strain, formulation, and rate challenged against [11]. The results described herein demonstrate these variations in fungal effectiveness when tested under laboratory and field conditions. For example, fungi are not universal in their ability to cause mortality, and some reports have shown that increased application rates of *Bb*-GHA may be needed against some tick species to provide suppression comparable to the same rate of *Mb*-F52 under controlled laboratory conditions. For example, mortality of *D. albipictus* larvae was significantly less when treated with *Bb*-GHA (30–41%) in comparison to *Mb*-F52 (82–99%) when the two strains were applied at the same rates [52]. In contrast, *Bb*-GHA killed *R. sanguineus* sensu lato nymphs faster and caused greater sporulation than *Mb*-F52 [54]. This difference might be attributed to *B. bassiana*’s propensity for profuse sporulation under more diverse conditions, where 91.5% of ticks that were infected with *Bb*-GHA sporulated compared with 77.3% of those infected with *Mb*-F52.

The performance of entomopathogenic fungi in field settings relies on the environmental conditions in which they are applied. Factors affecting the persistence and virulence of fungi within the environment include temperatures outside the general range of 23–28 °C, low humidity, ultraviolet radiation, precipitation, soil type and pH, and duff layer composition and nutrition [62,71,72]. Site (i.e., soil type, pH, leaf litter depth), weather, and microclimate conditions (i.e., soil moisture, temperature, humidity) were generally unreported in most studies mentioned herein, except for vegetation structure and composition. These factors play an integral role in explaining the efficacy of fungal treatments. For example, sandy, well-drained soils with acidic pH (5.1 to 4.5) and leaf litter and duff layers may have led to limited conidia contact with questing ticks that might have contributed the low performance of *Mb*-F52 in one report [73]. In another study, differences in interannual climatic conditions likely influenced differences in tick suppression using fungal biopesticides, where one year was hot and dry (drought-like) and the other was mild and wet [44]. The authors also indicated that factors related to the timing and delivery of fungal biopesticides to tick habitats are also a critical factor influencing efficacy and warrant further investigation (i.e., application rates and optimal sprayer volume and pressure). Applications of fungi early and later in the day may allow more optimal humidity conditions and protect from meteorological events such as wind [67]. Given some species like *I. scapularis* have been reported to be more active in the early morning and evening [74], the effects of application timing in relation to the diurnal activity of ticks should also be considered. Further evaluations against ticks should also focus on the performance of existing products and the development of novel delivery systems. Examples include the efficacy of fungal products that contain mixtures with reduced-risk pesticides such as neem oil and pyrethrins, or the development of auto-dissemination devices for use with trapping technologies like semiochemical and pheromone traps that take advantage of the tick’s host-seeking biology [75,76].

The requirements for appropriate environmental conditions (i.e., temperature, humidity) needed by fungal biopesticides create a challenge in reaching a level of suppression consistently comparable to that of synthetic acaricides and in reducing tick numbers to an acceptable level across a diverse range of conditions [11,43]. Synthetic chemical acaricides, with a few exceptions, consistently achieve a percent reduction in host-seeking nymphs in the field of 80–100% [7]. Chemical pesticides are the top choice made by pest management firms to suppress ticks, followed by natural or organic products, and bifenthrin continues to be the industry standard for management [4,9,41,42]. Natural-product-based acaricides containing cedar extracts are frequently used by commercial pest control firms [9]. Nootkatone has been extensively field tested and, with two exceptions, was shown to reduce questing nymphs by 41–100% [7]. However, like fungus-based biopesticides, natural products are more variable in their ability to reduce ticks and have less robust residual activity than synthetic pyrethroids (i.e., bifenthrin), often requiring multiple applications [9,41,73].

Ultimately, the intent of tick management is to reduce tick bites and the effects of parasitism (e.g., human disease) or to reduce the population of pathogen-infected vectors while considering effectiveness, environmental impact, and cost [77]. Ticks are long-lived arthropods and individuals of some species may spend 98% of their time off-host, free-living in the environment [78]. Research has demonstrated that it takes several weeks for significant tick mortality to occur. Arguably, mortality occurring after a few weeks using fungi is not too long considering the tick life span [64]. For mortality to occur, targeting host-seeking ticks with fungal applications when environmental conditions are conducive is critical (i.e., for fungal infection to suppress ticks in environments where there is a high-risk of tick–human contact to prevent tick-borne disease such as residential settings). For management of pathogen-vectoring ticks, applications should be applied prior to or at onset of activity (i.e., mid-April–May) in the northeastern to mid-Atlantic USA). For example, applications of synthetic pesticides close to the onset of nymphal activity can reduce the risk of exposure to host-seeking ticks and reduce the frequency of applications needed to maintain a high level of suppression (90%), thus saving on management costs from repeat applications, which were needed for *Mb*-F52 [42]. However, the cost and effects of multiple applications of minimal-risk products in the field on tick populations remain unclear [41], as do acceptable and realistic tick suppression thresholds [4,17], especially for microbial pesticides used in the context of ITM.

Use of fungal biopesticides has not gained traction with pest management companies. Pest management firms were surveyed in Pennsylvania, New Jersey, and New York USA and it was found they routinely charge $100–150 USD (34.9% of respondents) or $151–200 (56.1%) per visit to treat a 1 acre (0.4 ha) area for control of ticks with conventional synthetic pesticides [9]. Pathogenic fungi were not used by applicators and 88% indicated their lack of familiarity with the method as the reason why they lacked experience with its use, whereas only 3% indicated that the products were too expensive. Information on direct cost comparisons between chemical versus microbial controls of ticks is lacking. When residents in Lyme-disease-endemic communities (Connecticut, USA) were surveyed, most were not willing to spend over $100 USD on tick control [79]. In a more recent survey [80], 85% of respondents from Connecticut and Maryland, USA, were willing to pay for a yard treatment, yet still would not spend over $99 USD. Of those willing to pay, 95% would invest in natural pesticides and 63% would pay for chemical pesticides. This suggests there is a consumer demand for alternative control options for ticks if effective; however, public perception of control methods and willingness to pay for prevention would likely differ across states and should be assessed more closely.

A primary goal of the development of effective fungal biopesticides is to identify an entomopathogen as an active ingredient that economically produces stable propagules that can consistently manage the target pest under a variety of field conditions [81]. Quality control issues, the need for repeat applications, and relatively short shelf lives compared to synthetic acaricides drive up the associated costs in both production and application, both common drawbacks to fungal biopesticide use [73,82]. Improvements to the mass production of fungal biopesticides focus on propagule stability, infectivity and shelf life, and formulations and their delivery methods to optimize efficacy for targeted pests under a diversity of conditions [62,81,82,83].

## 5. Conclusions

It is likely that ticks will shift their ranges as the climate changes, continuing to impact the health of humans, domestic animals, and wildlife [5,84,85,86,87]. Under certain conditions, which generally remain unclear across tick habitats, the use of fungal biopesticides when used in combination with other reduction strategies might achieve results comparable to those achieved synthetic and natural-product-based acaricides if products were consistently available. The quest to identify efficacious, storage-stable, and environmentally persistent fungal biopesticide formulations for use against ticks should continue, given market attraction and adequate funding being allocated towards their development. Although product expansion has been slow for biopesticides for some of the reasons described herein, and in part because of registration complexities and the length of time from proof of concept to the marketing stage, there continues to be interest in production with growth potential as ITM/IPM approaches become more established across a diversity of pest management sectors [4,23,82,88,89,90]. Until alternative tick management strategies become widely available and more research on integrated approaches for other ticks of concern (i.e., *A. americanum* and *D. variabilis*) is conducted, management will rely primarily on synthetic, and to a lesser extent, natural-product acaricides [4,9]. The public is concerned about the negative impacts of synthetic pesticides on the environment and their health. As alternatives to synthetic pesticides become more readily available, it is likely that homeowners and commercial pest control firms will consider them if they are efficacious and economical [9,42,80].

## Figures and Tables

**Table 1:** Effects of commercial products containing *Metarhizium brunneum* (formerly *M. anisopliae*) on *Ixodes scapularis* evaluated under laboratory conditions.

Product/Strain	Life ^a^ Stage	Method	Exposure Rate and Time ^b^	Experiment Duration	Treatment Effect ^c^	Reference
Bio-Blast Biological Termiticide^™^ (strain ESC1)	A (u)	Spray	4 × 10^6–9^ c/mL	4 weeks	96% mortality at 10^9^ conidia/mL, LC_50_ = 4 × 10^7^ conidia/mL	[25]
	N (u)	Spray	10^6–9^ c/mL; 2.8 mL	4 weeks	70% mortality at 10^9^ conidia/mL, LC_50_ = 10^7^ conidia/mL	[34]
	N (u)	Topical with chemical pesticide	Permethrin (Bonide^®^) (0.1–1 ppm) at 2 µL then 10^7–9^ c/mL at 10 µL 1 min later	23 days	Mortality up to ~80% when ticks treated with 0.05 ppm permethrin and fungi at 10^8^ c/mL	[34]
	L, N	Treated cotton batting (nesting material)	10^8^ c/mL	72 h	Mortality of ticks dropped from *P. leucopus* was 75% in treated nest treatment	[39]
Tick-Ex^®^ EC (strain F52)	A (u)	Spray	2.6 × 10^2–6^ c/cm^2^ (3, 30, or 300 min)	4 weeks	8.3–100% mortality (3 and 30 min exposures); 0–100% (300 min exposure)	[40]
	N (u)	Spray	2.6 × 10^3–5^ c/cm^2^ (3 or 30 min)	4 weeks	10–14.2% mortality (3 min exposure); 6.1–70.8% mortality (30 min exposure)	[40]
	A (u)	Immersion	7.4 × 10^5–9^ c/mL (30 s)	4 weeks	8.3–100% mortality	[40]
	A (u)	Treated surface	2.6 × 10^5–8^ c/cm^2^ (3, 30, or 300 min)	4 weeks	8.3–100% mortality (3 min exposure); 0–100% (30 min exposure); 16.7–100% (300 min exposure)	[40]
	N (u)	Treated surface	2.6 × 10^3–6^ c/cm^2^ (3 or 30 min)	4 weeks	9.2–100% mortality (3 min exposure); 0–100% (30 min exposure)	[40]
Tick-Ex^®^G (strain F52)	A (u)	Broadcast	2.6 × 10^5–7^ c/cm^2^ (3, 30, or 300 min)	4 weeks	27.8–81.9% mortality	[40]
	N (u)	Broadcast	2.6 × 10^5–7^ c/cm^2^ (3, 30, or 300 min)	4 weeks	30.2–81.5% mortality	[40]

^a^ Life stage: A (adult); L (larvae); N (nymphs); (u) unfed. ^b^ c (in numerator) = conidia; cfu (in numerator) = colony-forming units; Time is the length of exposure to the treatment (if specified). ^c^ Treatment effect is cumulative to the specified evaluation time.

**Table 2:** Effects of commercial products containing *Metarhizium brunneum* (formerly *M. anisopliae*) on *Ixodes scapularis* evaluated in the field in residential lawn or woodland settings.

Product/Strain	Life ^a^ Stage	Method	Exposure Rate ^b^	Experiment Duration	Treatment Effect ^c^	Reference
Bio-Blast Biological Termiticide^™^ (strain ESC1)	A (u)	Spray	4 × 10^9^ c/mL; 1–1.5 L/100 m^2^	6 weeks	53% mortality among ticks collected from treated plots	[25]
	A (u)	Spray	10^8^ c/mL; 1–1.5 L/100 m^2^ (2×)	3 weeks	52% mortality among adults collected from field, 36% control in the field	[26]
	N	Spray	10^9^ c/mL; 1–1.5 L/100 m^2^	4 weeks	6–36% control in the field; 20–36% mortality in lab from field collected ticks post treatment	[34]
	L	Treated cotton batting (nesting material)	10 mL of 10^8^ c/mL (×10) at density of 9 boxes/ha	5 months-1 year	No significance difference in nymphal densities between areas with treated nest boxes and control; no effect on proportion of nymphs infected with *B. burgdorferi*	[39]
Met52^®^EC (strain F52)	N (u)	Spray	0.96 mL/m^2^ (year 1) 1.02 mL/ m^2^ (year 2) per arena	1 month; 2 years	10% knockdown control; 0% residual control (year 1), 41.3% and 29.8%, control respectively (year 2)	[41]
	N	Spray	10.6 mL/100 m^2^ (×3)	2.5 months	Target 90% suppression threshold inconsistently met	[42]
Tick-Ex^®^ EC (strain F52)	N	Spray	3.2 × 10^5^ and 1.3 × 10^6^ c/cm^2^ (×2)	3 and 5 weeks	87.1 and 96.1% fewer ticks collected from low- and high-rate sites, respectively, after 3 weeks, 53.2 and 73.8% reduction after 5 weeks, 36.4% nymphs collected infected with fungus	[43]

^a^ Life stage: A (adult); L (larvae); N (nymphs); (u) unfed. ^b^ c (in numerator) = conidia; cfu (in numerator) = colony-forming units; Time is the length of exposure to the treatment (if specified). ^c^ Treatment effect is cumulative to the specified evaluation time.

**Table 3:** Effects of commercial products containing *Beauveria bassiana* or *Metarhizium brunneum* (formerly *M. anisopliae*) on *Ixodes scapularis* evaluated in combination with other reduction strategies or as part of an integrated tick management program in residential lawn or woodland settings.

Product/Strain	Life ^a^ Stage	Method	Exposure Rate ^b^	Experiment Duration ^c^	Treatment Effect ^d^	Reference
*Beauveria bassiana*						
BotaniGard^®^ ES (strain GHA)	N	Spray + wood chip barrier + lawn perimeter debris removal	9.9 × 10^11^ c/100 m^2^ (×2)	3 months; 2 years	Without wood chip barrier: 74.5% (year 1) and 55.2% (year 2) tick reduction; with barrier: 88.9% (year 1) 55.1% (year 2) tick reduction	[44]
Naturalis^®^ T&O (strain ATCC 74040)	N	Spray + wood chip barrier + lawn perimeter debris removal	2.2 × 10^9^ c/100 m^2^ (×2)	3 months; 2 years	Without wood chip barrier: 83% (year 1) and 38% (year 2) tick reduction; with barrier: 90% (year 1) 56% (year 2) tick reduction	[44]
*Metarhizium brunneum*						
Met52^®^EC (strain F52)	L, N	Spray + deer reduction + fipronil bait box	5.5 × 10^9^ cfu/g 0.63–0.96 mL/m^2^ (×2)	3 months; 2 years	53% reduction in the potential to encounter a questing nymph infected with a pathogen in fungus/bait box treatment; 90% reduction in immature ticks parasitizing *P. leucopus* in the three combined treatments, 93% reduction in fungus/bait box treatment	[45]
	N	Spray + deer reduction + fipronil bait box	5.5 × 10^9^ cfu/g 0.63–0.96 mL/m^2^ (×2)	3 months; 4 years	78–95% reduction in questing nymphs in fungus/bait box treatment each year; 66% reduction in the potential to encounter a pathogen-infected questing nymph observed in one year	[46]
	L	Spray + deer reduction + fipronil bait box	5.5 × 10^9^ cfu/g 0.63–0.96 mL/m^2^ (×2)	3 months; 3 years	94% reduction in pathogen-infected larvae parasitizing *P. leucopus* in fungus/bait box treatment; 85% reduction in the three combined treatments	[47]
Tick-Ex^®^ EC (strain F52)	N	Spray with botanical pesticide	2.8 × 10^9^ c/m^2^ with 0.05% nootkatone	3 months; 3 years	50% control for one week and no control for the remainder of the season	[48]
	N	Spray + lawn perimeter debris removal	2.5 × 10^11^ c/100 m^2^ (×2)	3 months; 1 year	55.6% tick reduction from lawn perimeter treatment; 84.6% reduction from woodland treatment	[44]

^a^ Life stage: A (adult); L (larvae); N (nymphs). ^b^ c (in numerator) = conidia; cfu (in numerator) = colony-forming units. ^c^ Duration of sampling effort within a season; year after fungal application. ^d^ Treatment effect is cumulative to the specified evaluation time.

**Table 4:** Effects of commercial Met52^®^ products containing *Metarhizium brunneum* strain F52 (formerly *M. anisopliae*), or the isolate, on ixodid ticks evaluated under laboratory conditions (unless indicated otherwise).

Tick Species	Material ^a^	Life ^b^ Stage	Method	Exposure Rate and Time ^c^	Experiment Duration	Treatment Effect ^d^	Reference
*Amblyomma Americanum* (Lone star tick)	P-EC	A, N	Spray (woodlands)	10.6 mL/100 m^2^ (3×)	2.5 months	Target 90% suppression threshold inconsistently met for nymphs, low suppression for adults	[42]
*Boophilus microplus* (Cattle tick)	P-EC	A (f)	Immersion	1 × 10^6–8^ cfu/mL (30 s)	12 days	100% mortality; egg mass weight reduced by ~80%	[49]
	P-EC	L (f)	Immersion	1 × 10^6–8^ cfu/mL (30 s)	1 week	Nearly 100% at 10^8^	[49]
*Dermacentor albipictus* (Winter tick)	IS-EC	L (u)	Immersion	1.6 × 10^7^ c/mL (1 min)	15 days	LT_50_ = 3.7; 100% mortality	[50]
	IS-EC	L (u)	Immersion	1.3 × 10^7^ c/mL (1 min)	10 days	83.1% mortality for hatching-age; 86.8% for 14-day-old and 81.1% for 5-months-old	[51]
	IS-EC	E	Immersion	1.3 × 10^7^ c/mL (1 min)	10 days	71.5% failed to hatch treated at oviposition; 67.4% failed to hatch 14 days after oviposition	[51]
	P-EC	L (u)	Spray	1 × 10^5^ and 2 × 10^5^ c/cm^2^	9 days	94% and 98% mortality	[52]
	P-EC	L (u)	Spray	2.4 × 10^7^ c/0.007 m^2^	18 weeks and 3 weeks	~39% mortality when treated during summer quiescence and ~71%, during fall questing	[38]
*Dermacentor albipictus* (Winter tick)	IS-G	L (u)	Spray	1 × 10^6–8^ c/mL	3 weeks	82–99% mortality	[52]
	IS-G	L (u)	Immersion	1 × 10^6–8^ c/mL (1 min)	3 weeks	46.7%, 100% and 100% mortality	[38]
	P-G	L (u)	Broadcast	1–4 × 10^7^ c/0.002 m^2^ dish	3 weeks	72–89.3% mortality	[38]
	P-G	L (u)	Broadcast	1 × 10^8^ c/0.007 m^2^	18 weeks and 3 weeks	~95% mortality when treated during summer quiescence and ~1%, during fall questing	[38]
*Dermacentor reticulatus* (Ornate cow tick)	IS-G	A (u)	Immersion	10^2–8^ cfu/mL (3 min)	3 weeks	Up to 100% mortality; LC_50_ = 2.0 × 10^6^ cfu/mL (females)	[53]
*Ixodes ricinus* (Castor bean tick)	IS-G	A (u)	Immersion	10^2–8^ cfu/mL (3 min)	3 weeks	Up to 100% mortality; LC_50_ = 1.6 × 10^6^ cfu/mL (females).	[53]
*Rhipicephalus sanguineus* (Brown dog tick)	P-EC	N	Treated surface	1 × 10^9^ c/mL (1.48 × 10^7^ c/cm^2^)/pack (60 min)	4 weeks	>80% mortality; 77.3% of infected ticks sporulated	[54]

^a^ EC, emulsifiable concentrate; G, granular; IS, strain was isolated from product then tested; P, product was tested. ^b^ Life stage: A (adult); E (egg); L (larvae); N (nymphs); (f) fed; (u) unfed. ^c^ c (in numerator) = conidia; cfu (in numerator) = colony-forming units; Time is the length of exposure to the treatment (if specified). ^d^ Treatment effect is cumulative to the specified evaluation time.

**Table 5:** Effects of the commercial *Beauveria bassiana* product BotaniGard^®^ (strain GHA), or the isolated strain, on ixodid ticks evaluated under laboratory and field conditions.

Tick Species	Material ^a^	Life ^b^ Stage	Method	Exposure Rate and Time ^c^	Experiment Duration	Treatment Effect ^d^	Reference
Laboratory							
*Amblyomma americanum* (Lone star tick)	P-ES	A (u)	Immersion	10^8^ c/mL (10 s)	26 days	Treated ticks survived a mean of 7.2 days	[66]
*Dermacentor albipictus* (Winter tick)	IS	L (u)	Spray	1 × 10^6–8^ c/mL	21 days	30–41% mortality	[52]
*Dermacentor reticulatus* (Ornate cow tick)	IS-WP	A (u)	Immersion	10^2^–10^8^ cfu/mL (3 min)	3 weeks	Up to100% mortality; LC_50_ = 6.8 × 10^3^ cfu/mL	[53]
*Ixodes ricinus* (Castor bean tick)	IS-WP	A (u)	Immersion	10^2^–10^8^ cfu/mL (3 min)	3 weeks	Up to 100% mortality; LC_50_ 3.3 × 10^6^ cfu/mL for adult females	[53]
*Rhipicephalus sanguineus* (Brown dog tick)	P-ES	N	Treated surface	1 × 10^9^ c/mL (1.48 × 10^7^ c/cm^2^)/pack (60 min)	4 weeks	>90% mortality	[54]
Field							
*Amblyomma americanum* (Lone star tick)	P	A (u)	Immersion then placed in arenas	10^8^ c/mL (1 s)	2 weeks	Up to 96% mortality	[67]
*Hyalomma lusitanicum* ^e^	P	All stages	Sprayed host burrows	2.43 × 10^8^ c/mL (spring)	30 and 60 days	78.63% and 63.28% parasitism reduction on rabbits in spring, 35.72% and 29.01% in summer	[68]

^a^ ES, a emulsifiable concentrate; IS, strain was isolated from product then tested; P, product was tested; WP, wettable powder. ^b^ Life stage: A (adult); E (egg); L (larvae); N (nymphs); (f) fed; (u) unfed. ^c^ c (in numerator) = conidia; cfu (in numerator) = colony-forming units; Time is the length of exposure to the treatment (if specified). ^d^ Treatment effect is cumulative to the specified evaluation time. ^e^ During summer, *Hyalomma lusitanicum* was dominant species (79.95%), others included *Rhipicephalus pusillus* (11.08%), *Haemaphysalis hispanica* (8.94%), *Ixodes ventalloi* (0.02%), and *Dermacentor marginatus* (0.01%).

## Data Availability

Not applicable.
